# Optimizing depression detection in clinical doctor-patient interviews using a multi-instance learning framework

**DOI:** 10.1038/s41598-025-90117-w

**Published:** 2025-02-24

**Authors:** Xu Zhang, Chenlong Li, Weisi Chen, Jiaxin Zheng, Feihong Li

**Affiliations:** 1 School of Software Engineering, Xiamen University of Technology, Xiamen, 361024 China; 2 School of Computer and Information Engineering, Xiamen University of Technology, Xiamen, 361024 China

**Keywords:** Deep learning, Multiple instance learning, Depression detection, Model Ensemble, Interview data, Machine learning, Depression

## Abstract

In recent years, the number of people suffering from depression has gradually increased, and early detection is of great significance for the well-being of the public. However, the current methods for detecting depression are relatively limited, typically relying on the self-rating depression scale (SDS) and interviews. These methods are influenced by subjective or environmental factors. To improve the objectivity and efficiency of diagnosis, deep learning techniques have been applied to the field of automatic depression detection (ADD), providing a more accurate and objective approach. During interviews, transcribed interview data is one of the most commonly used modalities in ADD. However, previous studies have only utilized response texts or selected question–answer pairs, resulting in information redundancy and loss. This paper is the first to apply the multiple instance learning (MIL) framework to the field of textual interview data, aiming to overcome issues of inadequate text representation and ineffective information extraction in long texts. In the MIL framework, each instance undergoes an independent feature extraction process, ensuring that the local features of each instance are fully captured. This not only enhances the overall text representation capability but also alleviates the issue of sample imbalance in the dataset. Additionally, this paper improves upon previous aggregation strategies by introducing two hyper-parameters to accommodate the uncertainties in the field of text sentiment. An ensemble model of MT5 and RoBERTa (referred to as multi-MTRB) was constructed to extract features from each instance and output confidence scores indicating the presence of depressive information in the instances. Due to the unique design of the MIL framework, the proposed method is highly interpretable and is able to identify specific sentences that identify people from depressed patients, while introducing LIME techniques to provide more in-depth interpretation of negative instance sentences. This provides a promising approach for depression detection in the context of text interview data patterns. We evaluated the proposed method on DAIC-WOZ and E-DAIC datasets with excellent results. The F1 score is 0.88 on the DAIC-WOZ dataset and 0.86 on the E-DAIC dataset.

## Introduction

In the current global public health field, depression has become a topic of great concern. According to statistics from the World Health Organization (WHO)^1^, depression has an extremely high incidence rate worldwide, with an estimated 350 million people suffering from depressive disorders globally. On average, 1 in 20 people has experienced or is currently experiencing the torment of depression. The prevalence of this mental illness makes it one of the significant factors affecting people’s mental health. In the adolescent population, the prevalence of depression is on the rise. It is estimated that by 2024, the number of adolescents with depression globally will reach an alarming 630 million, accounting for 15% of the global adolescent population. Depression during adolescence can lead to recurrence in later life and is often accompanied by other adverse outcomes such as anxiety disorders, suicidal behavior, and substance abuse^2^. This trend highlights the severity of depression among the younger generation and reveals the urgent need for increased attention to adolescent mental health issues.Depression patients often face multiple challenges. Issues such as strong stigma, lack of disease education, and difficulty in accessing medical care make it hard for patients to receive timely and effective treatment. Research^3^ shows that early identification and support of individuals with depressive tendencies can significantly reduce the likelihood of developing depression. Therefore, improving the accuracy and reliability of depression detection technology is crucial.

Text plays a crucial role in the diagnosis of depression, serving not only as a carrier of information but also as a direct reflection of the speaker’s emotions and thoughts. In recent years, with the continuous development of natural language processing technology, many researchers have utilized text to conduct depression detection studies. Research shows^4^ that there are significant differences in word choice and expression between individuals with depression and healthy individuals, providing new clues and evidence for the early detection of depression. Past studies have revealed that our language use can profoundly reflect our personality traits, social status, emotional fluctuations, and mental health conditions^5^. Burdisso et al.^6^ constructed a two-layer GCN model that utilized the relationships between documents and words. By establishing a heterogeneous graph containing word nodes and document nodes and defining different types of edges, they learned representations for text classification tasks. Qureshi et al.^7^ conducted experiments on benchmark datasets and demonstrated significant performance improvements compared to single-task and multi-task methods without emotional awareness. Text feature representation based on pre-trained models holds great potential in the field of depression detection, especially in handling sparse data and generating robust semantic representations. Xue et al.^8^ used the pre-trained BERT model to extract sentence-level embeddings, achieving an F1 score of 0.85. However, their approach lacked deep exploration of emotional and semantic information and did not fully utilize text features. Zhang et al.^9^ introduced a multi-level depression state detection method based on fine-grained prompt learning (MDSD-FGPL), which is more valuable for the early detection and timely intervention of depression.

Although many advanced methods are currently being used in depression detection and have achieved good results, there are still some challenges. First, in the diagnosis of depression, the diagnosis comes from a prolonged exchange between the doctor and the patient. In the case of long text data such as clinical interviews, previous studies have generally treated long texts similarly to short texts, which are entered and computed as a whole. Mao et al.^10^ used a sliding window technique to zero-pad short sentences; Guo et al.^11^ extracted 10 thematic question–answer pairs from the interview data. However, such processing tends to overlook local information and cause some information loss. Second, the manifestations and symptoms of depression are diverse and may vary due to individual differences, cultural backgrounds, social environments, and other factors^12^. Traditional single models may find it difficult to capture and analyze the complex features in such diverse data. Third, previous machine learning models often lack interpretability when providing predictive results, making it difficult for doctors to understand how the model makes decisions and what factors contribute to a patient’s depression. This lack of interpretability is particularly evident in interview data, as it usually contains a large amount of complex textual information involving the patient’s personal experiences, emotional states, social environments, and other aspects. Traditional models struggle to parse this multidimensional information and cannot provide specific explanations to help doctors understand the patient’s condition.

**Fig. 1:**
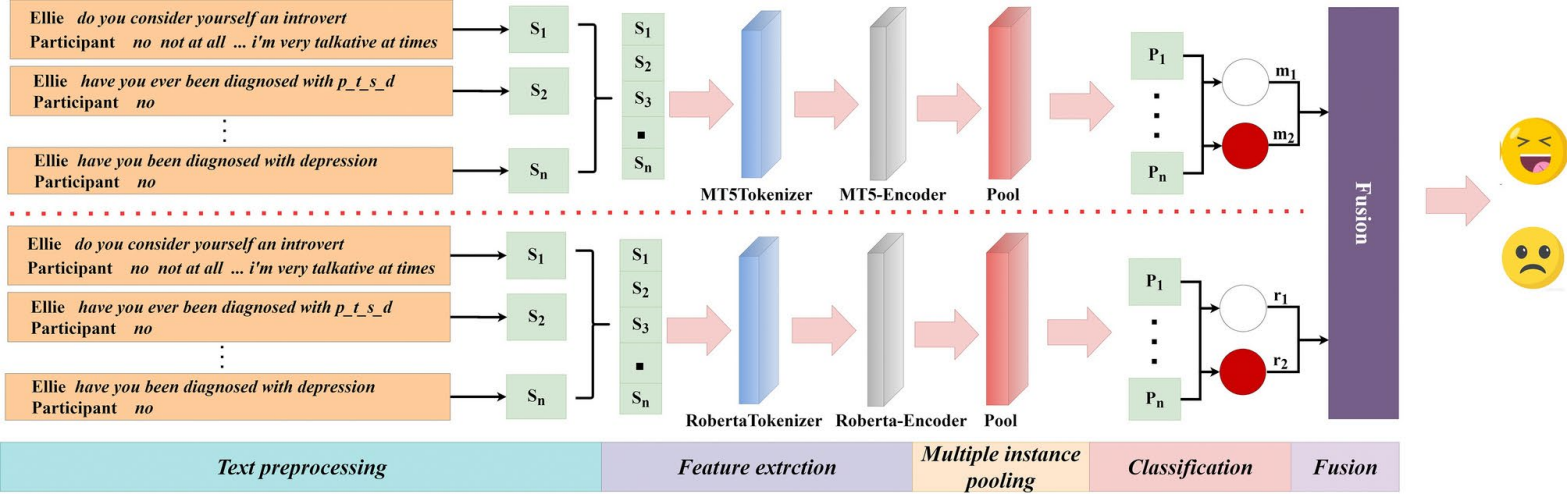
The overall framework of the proposed model named Multi-MTRB, which consists of two depression detection branches, namely Multi-MT5 and Multi-RoBERTa.

Addressing the three core challenges faced by the field of automatic depression detection (ADD), the research objectives of this paper primarily encompass three aspects. Firstly, this study aims to introduce the multi-instance learning (MIL) method into the processing of depression interview data, to achieve precise segmentation of participants’ responses. This method will deeply explore the local information within the interview data, effectively extracting key features closely related to depression, laying a solid foundation for subsequent depression detection. Secondly, this study plans to integrate the concept of multi-instance learning into the pre-trained models MT5 and RoBERTa, innovatively constructing the Multi-MT5 and Multi-RoBERTa models. Through the deep fusion of these two models, they will be able to more keenly capture and analyze complex features related to depression, further enhancing the accuracy and depth of detection. Thirdly, this study will improve the aggregation strategy of multi-instance learning by introducing two hyperparameters to address the uncertainty in the field of text sentiment. By utilizing the multi-instance learning framework to decompose long text data into multiple instances (such as specific answers to each question), and analyzing the emotional information and semantic features of each instance, the model will not only be able to identify which answers contain depressive information but also quantify the depressive information content in these answers, providing a finer-grained explanation. To achieve the aforementioned objectives, this paper develops a multi-level depression detection method based on multi-instance fusion, with the primary goal of improving the accuracy and reliability of depression detection. As illustrated in Fig. 1, this method aims to facilitate precise identification and early detection of depression through comprehensive analysis and in-depth exploration of multi-level information within interview data.

In summary, the main contributions of our paper are as follows:

1. For long text multi-round dialogue data, we introduce multi-instance learning (MIL) to segment the data, addressing the issues of inadequate text representation and inability to extract effective information in the task of depression classification in multi-round dialogue texts.
2. In the field of depression detection research, we propose the Multi-MT5 and Multi-RoBERTa models and fuse these two models, referred to as Multi-MTRB. This aims to more effectively capture and analyze the complex features related to depression from diverse data, thereby further improving the accuracy and reliability of depression detection.
3. Improving the MIL by introducing two hyperparameters to change the MIL’s aggregation and strategy. Using the improved MIL, we can determine which specific responses best reflect a patient’s depressive symptoms. Doctors not only have access to the model’s predictions, but also can understand how the model arrived at those results. This transparency and interpretability is particularly important for clinical applications, providing more effective treatment options for patients.

## Related work

### Text-based depression detection

With the development of science and technology, the healthcare industry is entering the era of artificial intelligence. AI can assist in diagnosis and treatment, alleviating the pressure on doctors when analyzing data. AI is gradually transforming traditional diagnostic and treatment models and is expected to address the aforementioned shortcomings^13^. In clinical practice, the manifestations of depression in patients are often complex and diverse, with their emotional expressions spanning multiple modalities. Huiting Fan et al.^14^ proposed a Transformer-based multimodal feature enhancement network for integrating video, audio, and remote photoplethysmography (rPPG) signals to improve the accuracy of depression detection. Ashutosh Anshul et al.^15^ established a COVID-19 dataset and achieved good results in detecting depression through social media data during the COVID-19 pandemic by combining text, user-specific information, and image analysis. Although various modalities provide valuable perspectives for understanding depression, verbal expression still holds a special and important position in depression research. This is because words can not only directly express thoughts and emotions but also record patients’ inner thoughts and feelings, providing us with the possibility to deeply analyze and understand the psychological state of depression patients. Therefore, in subsequent research and discussions, we will focus on the application and potential of verbal expression in the detection, diagnosis, and treatment of depression.

Text is a direct medium for expressing thoughts and emotions, and the changes in language during interviews are very subtle^10,16,17^. Therefore, many researchers have explored the differences in text expression between depression patients and healthy individuals from a textual perspective. Text-based depression sentiment analysis methods^18,19^ mainly include emotion dictionary-based methods, machine learning-based methods, and deep learning-based methods.

Emotion dictionary-based text sentiment classification methods require the manual construction of an emotion corpus, where the emotional words, their polarities, and other attributes form an emotion dictionary. During sentiment recognition, the computer compares the words in the input text with the emotional words in the corpus or calculates the distance to find matching emotional words and their polarities, which represent the sentiment tendency expressed by the input text. Karmen C et al.^20^ constructed a small lexicon containing depression symptoms, but this lexicon has not been widely used because building such dictionaries requires a large amount of data and certain professional knowledge.

Machine learning-based sentiment analysis methods involve using large amounts of labeled or unlabeled corpora, employing statistical machine learning algorithms to extract features, and then performing classification. These methods yield better results and have broader application scenarios. Social networks, as platforms for the public to express opinions, and emotions, and share information, play a crucial role in sentiment analysis. Chiong R et al.^21^ designed a feature extraction method combining Bag of Words (BOW) and N-gram techniques and studied machine learning classifier models such as logistic regression (LR), SVM, decision tree (DT), random forest (RF), and XGBoost (GB). They trained and validated the model performance using several datasets from Twitter, Facebook, Reddit, and electronic diaries. The experiments showed that the classifier based on LR outperformed other classifiers across several datasets.

Deep learning is an important branch of machine learning and a significant revolution in the field. It simulates the mechanisms of the human brain by establishing deep neural networks, which can automatically mine latent features from data, thus performing tasks such as sentiment recognition. Amanat A et al.^22^ used a large Twitter dataset obtained from the Kaggle website to construct an RNN-LSTM model to predict depression from text. They compared the RNN-LSTM model with Naive Bayes, SVM, CNN, and Decision Tree algorithms, and the results showed that the proposed framework achieved higher accuracy, precision, recall, and F1 scores. Vandana et al.^23^ proposed a hybrid model for depression detection based on deep learning algorithms, using text features to train a CNN model. The results indicated that deep learning is a better solution for depression detection.

Most of the research on depression detection to date has been based on short texts from social media platforms such as Twitter and Weibo. However, due to the anonymity of these media, the data quality is relatively poor, and healthy individuals may disguise themselves as patients with mental disorders. To overcome these issues, this paper adopts the text modality of clinical interview data. Clinical interview data typically involve multiple rounds of questions and answers and thus can be defined as long text data. Unlike previous approaches, this experiment retains the paragraph structure of long texts during the data input stage and employs the concept of Multi-Instance Learning (MIL). Specifically, we treat the long text as a bag, with each paragraph considered an instance. This approach allows us to preserve the overall structure of the text while independently analyzing each paragraph, thereby improving the model’s detection accuracy.

### Multiple instance learning

Multiple instance learning (MIL) is a variant of supervised learning designed to solve the classification problem when individual instance labels are unavailable or difficult to obtain^24^. It effectively addresses the problem of distinguishing "coarse-labeled" objects when fine-grained labels are missing^25,26^. Currently, MIL problems can be divided into two categories: bag-level classification and instance-level classification^27,28^. In bag-level classification, the goal is to predict the label of the entire bag based on all the instances within the bag^29^. Each bag contains multiple instances but has only one label. Common applications include drug activity prediction, image classification, and text classification. In these applications, the model makes an overall judgment by aggregating the features of all instances within the bag. For example, in an image classification task, the entire image can be considered a bag, and multiple regions (instances) within the image are used to predict the overall category of the image.

In instance-level classification, the goal is to classify each instance within the bag, rather than just predicting the label of the bag^30^. This approach allows for more fine-grained analysis, enabling the identification of specific instances within the bag that have a significant impact on the bag’s label. For example, in medical diagnosis, multiple test results (instances) of a patient can be considered a bag, and instance-level classification can help identify which specific test results most accurately reflect the patient’s health condition.

Combining deep features, multi-instance learning (MIL) has demonstrated strong representational capabilities in recent research^27^. Zhao Y et al.^31^ proposed an embedded space MIL image classification method based on feature selection and graph convolutional networks. Struski et al.^32^ introduced the ImpoMIL method for MIL in medical image classification, enhancing the interpretability of MIL models. Liu et al.^33^ proposed the SMITL method based on MIL, achieving secure knowledge transfer from source tasks to target tasks. Angelidis et al.^34^ introduced a neural network model based on MIL, MILNET, which achieved excellent performance in fine-grained sentiment analysis tasks, providing new insights and methods for research on MIL in the field of sentiment analysis.

Since most interview texts contain numerous paragraphs, and these paragraphs are often unrelated, these texts do not consistently contain depressive information throughout. The majority of interviews will include normal conversation paragraphs or sections, and if an entire interview text is treated as a whole, the normal conversation parts can significantly impact the judgment of depression. Moreover, the numerous paragraphs in long texts add considerable difficulty to feature extraction. Therefore, this paper continues the conclusions drawn from previous research and uses bag-level classification in multi-instance learning (MIL) to address the classification task of textual interview data.

## Methods

### Dataset

The dataset used in our study was a corpus of clinical interviews collected by the the University of Southern California (USC) Institute for creative technology: the distress analysis interview corpus with Wizard-of-Oz (DAIC-WOZ) and the Extended DAIC-WOZ(E-DAIC). The DAIC-WOZ database is part of the Depression Analysis Interview Corpus (DAIC), which consists primarily of transcripts of clinical interviews. This corpus consists of 189 clinical interview recordings and transcripts, as well as facial features of 189 participants. The dataset has been divided into training, validation, and test sets, with the training set containing 107 participants, the validation set including 35 participants, and the test set comprising 57 participants^35^. Within the test set, there are 33 non-depressed and 14 depressed individuals. All interviews have been diligently transcribed into English. Overall, the DAIC-WOZ dataset encompasses 189 clinical interviews, including 107 for training, 35 for validation, and 47 for testing. The PHQ-8 total scores range from 0 to 24, with scores above 10 being indicative of depression. The Extended DAIC-WOZ(E-DAIC) serves as an extended version of the DAIC-WOZ, featuring semi-clinical interviews designed to aid in diagnosing psychological distress conditions, such as anxiety and depression^36^. This dataset is also divided into training, validation, and test sets. It contains 163 participants in the training set, with 56 participants each in the validation and test sets. Of those in the test set, 39 are non-depressed and 17 are diagnosed with depression.

### Multiple instance learning framework

This paper introduces a multi-instance learning (MIL) framework for the task of depression detection. In this context, positive samples (depression patients) are often fewer compared to negative samples (non-depression patients), leading to an imbalance in the dataset. The MIL framework, by considering multiple instances, helps to alleviate this sample imbalance issue. Previously, Multiple Instance Learning (MIL) has been widely applied in the field of computer vision to address multi-instance problems in images or videos. Applying the MIL framework to the domain of text interview data, particularly for the task of Automatic Depression Detection (ADD), is a relatively novel and unique attempt. This application expands the scope of MIL and demonstrates its potential in Natural Language Processing (NLP) and sentiment analysis. We also introduce two hyperparameters α and β to mitigate the uncertainty within the field of text sentiment analysis, which arises from participants’ inability to properly express their emotions or providing incorrect responses. Our approach uses bag-level predictions designed to predict patients’ labels as depressed or non-depressed.

**Fig. 2:**
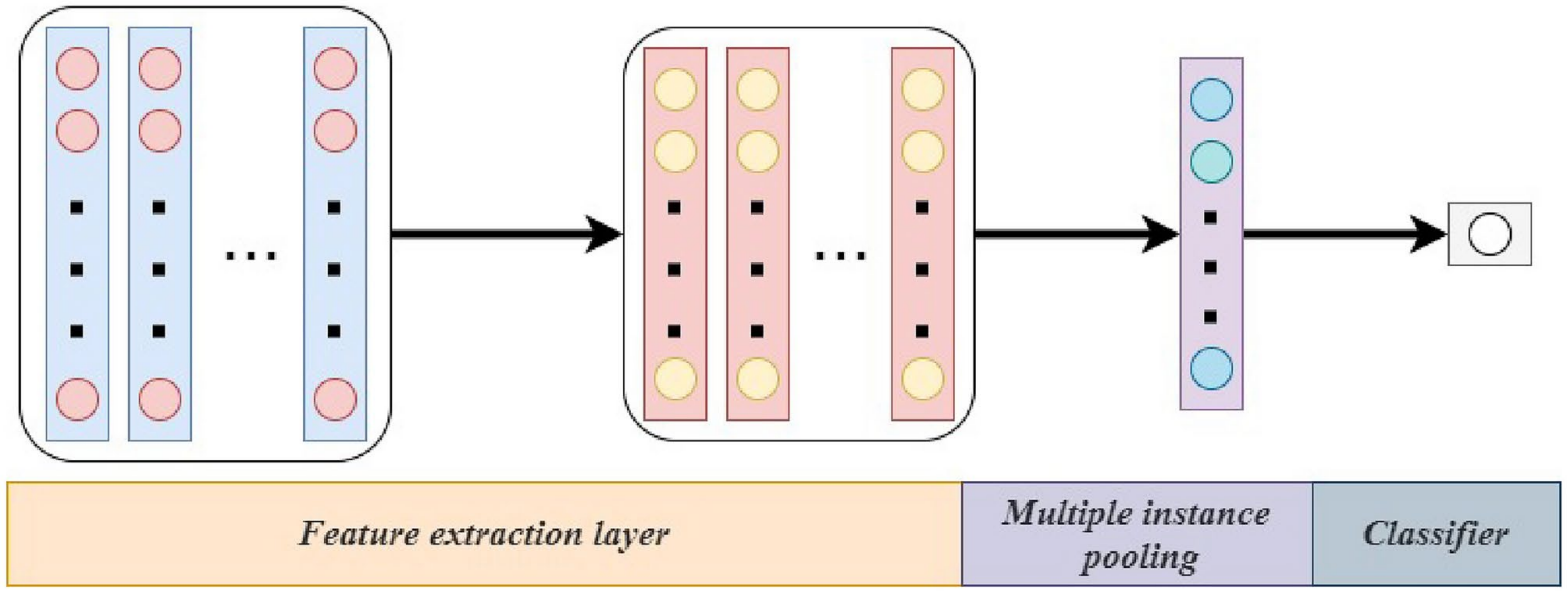
Deep multiple instance learning network architecture diagram. Here, the small circles represent “instances”, and the rectangles represent “bags”.

Deep learning techniques are widely applied in the field of Multi-Instance Learning. Although various network models have been developed, they still share some common basic structures. As shown in Fig. 2, these typically include three main parts: the feature extraction layer, the multi-instance pooling layer, and the classification layer^37^. These parts are stacked together in a specific order. The feature extraction layer usually consists of multiple layers and is used to extract useful feature information from the input data. The subsequent multi-instance pooling layer aggregates the extracted instance features, to integrate multiple instances into a single instance to facilitate subsequent classification or prediction tasks. It is noteworthy that the main differences between different deep MIL networks often lie in the pooling methods they employ. These differences not only affect the performance of the networks but also reflect the researchers’ various understandings and approaches to addressing MIL problems.

In the setting of the MIL (multiple instance learning) framework, we adopt a strategy where the responses of individuals to all inquiries are treated as a “bag,” with each specific response to a question forming an “instance” within this “bag.” When all instances in the bag reflect normal conversational information, we classify it as a “negative bag,” indicating the absence of depressive information. Conversely, as soon as one or more instances in the bag show signs of depressive tendencies, the entire bag is labeled as a “positive bag,” indicating the possibility of depressive information. Specifically, a “bag” is labeled as positive if it contains at least one positive instance; otherwise, it is labeled as negative. Taking binary classification as an example, consider the existence of a “bag” denoted as $b = \{ (x_{1} ,y_{1} ), \ldots ,(x_{n} ,y_{n} )\}$. Where the instance $x_{i} \in x$ with the classification label $y_{i} = c(x_{i} ) \in \{ 0,1\}$, the label of the bag $Y_{b}$ can be calculated using the following formula:

1 $$Y_{b} = \left\{ {\begin{array}{*{20}c} {0,{\text{if}}\sum\limits_{i = 1}^{n} {y_{i} = 0} } & {} \\ {1,\;\;{\text{otherwise}}} & {} \\ \end{array} } \right.$$

The label of the “bag” can be calculated using transformation operation $f$ and permutation-invariant transformation operation $g$, as shown below:

2 $$Y_{b} = g(f(x_{1} ), \ldots ,f(x_{n} ))$$

Therefore, in multiple instance learning, different implementations of $f$ and $g$ are commonly modeled, primarily through the following two methods.

1. Instance-based methods. $f$ represents the instance classifier used to calculate the predicted scores of instances for each class. The bag score is then predicted by aggregating the scores of all instances within the bag using $g$.
2. Embedding-based methods. $f$ represents the instance encoder, used to map instances to low-dimensional representation vectors. The bag-level vector is then formed by aggregating all instance vectors within the bag using $g$, and the final score of the bag is predicted.

**Fig. 3:**
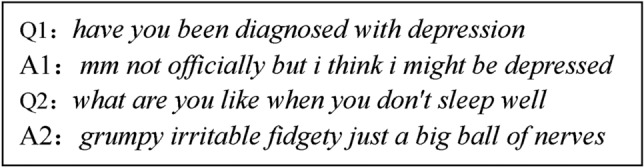
Two question-and-answer pairs of participant 302.

However, we must recognize that factors such as the subjects’ emotional state, subjective awareness, and sensitivity to the questions can all influence their responses, sometimes leading to expressions that resemble “depression.” Most importantly, one of the reasons early depression is not easily detected is that the thoughts and behaviors are similar to those of normal individuals. For example, Participant 302 exhibited some emotions similar to those of depression patients when faced with certain questions. The following figure shows the selected question-and-answer pairs:

From the questions and answers in Fig. 3, participant 302 does exhibit some emotions. In fact, this participant does not have depression, but they feel that they do, which can lead to strong depressive information in their response text. We cannot simply judge them as a depression patient based on these two responses alone. We should not easily determine the subject as a depression patient solely based on depressive information from a single instance within the bag; instead, a more comprehensive judgment should be made based on multiple responses.

**Fig. 4:**
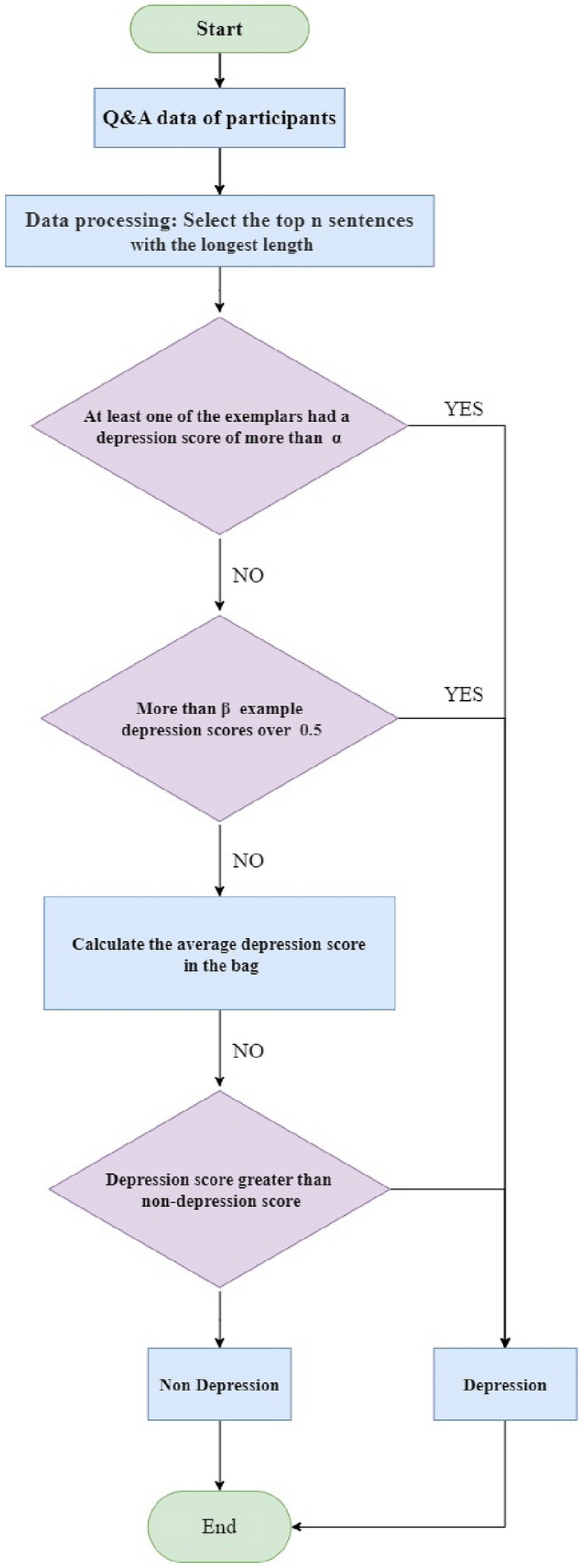
Flowchart for determining depression in instances.

To improve this judgment logic, we have refined the traditional MIL judgment logic, as shown in (Table 1). We have established the following two criteria for interview data: First, we will diagnose the participant as having depression only if more than half of the instances within a bag exceed a preset threshold $\alpha$ on the depression score. Second, when the depression score of an instance exceeds 0.5, we define that instance as a depressive instance (Fig. 4). If there are more than $\upbeta$ depressive instances within the bag, we will also make a diagnosis of depression. These improvements aim to enhance the accuracy and reliability of the diagnosis, ensuring that the judgment of participants is more scientific and fair. Below is the flowchart of the MIL judgment logic:

**Table 1:**
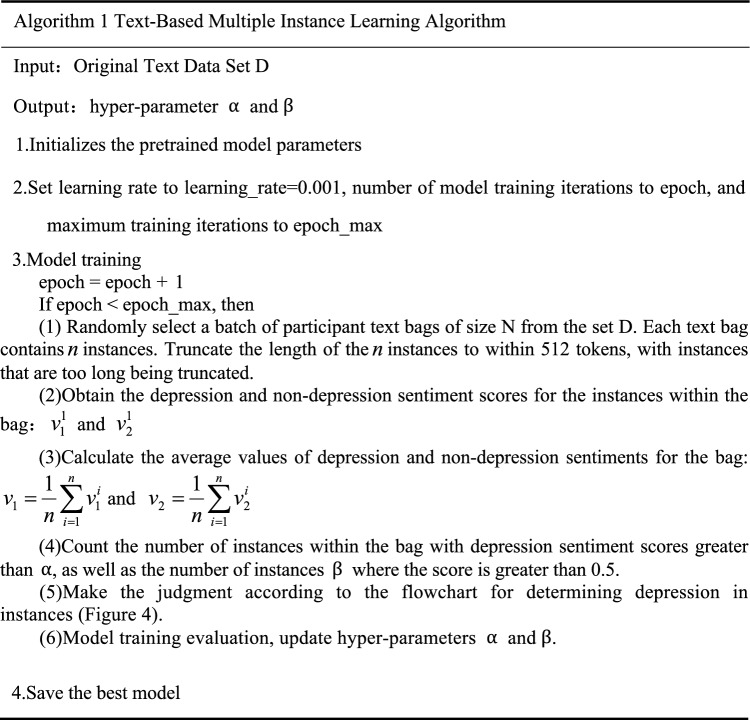
Text-based multiple instance learning algorithm.

### The multi-MT5 model

The MT5 model, as a multilingual text-to-text transfer transformer based on the Transformer architecture, has demonstrated powerful capabilities in text classification tasks^38^. By pretraining on a large-scale multilingual dataset, the MT5 model is able to capture cross-lingual textual features and learn the mapping from text input to text output. Therefore, compared to the T5 model, the MT5 model can capture richer contextual information, which is crucial for understanding the subtle emotional changes in depression interviews. For depression interview data involving different languages or dialects, ensuring that the model can understand and analyze various expressions is also particularly important.

In text classification tasks, the MT5 model can be fine-tuned using labeled data specific to the task, adjusting the model parameters to meet different classification needs. By inputting text into the encoder part of the MT5 model, the model can generate vector representations of the text; these vector representations are then fed into a classification layer (such as a softmax layer) to output the category labels to which the text belongs.

For the obtained text data of the respondent sample ${\text{S}}_{\text{i}}$ we first perform an ordered segmentation based on timestamps, and then arrange all involved question-and-answer sessions in the chronological order of their occurrence:

3 $$S_{i} = \{ Q_{i,1} ,A_{i,1} , \ldots A_{i,m} , \ldots ,Q_{i,n} ,A_{i,1} , \ldots ,A_{i,m} \}$$

The variable $n$ represents the number of questions, which may vary depending on the package. The variable $A_{i,1}$ refers to the first response to the $i$ question, where one question may correspond to multiple responses. We aggregate all responses to the same question. Therefore, $S_{i}$ can be further expressed as:

4 $$S_{i} = \{ S_{i,1} ,S_{i,2} , \ldots S_{i,n} \}$$

First, we input $S_{i,1}$ into the MT5 tokenizer to obtain the tokenized result $MT_{i}$. Next, we use the MT5 encoder to obtain the encoded result $E_{i}$:

5 $$E_{i} = MT5 - Encoder(Mt5Tokenizer(S_{i} ))$$

### The multi-RoBERTa model

The RoBERTa pre-training model, as an enhancement of the BERT (Bidirectional Encoder Representations from Transformers) model, was launched by Facebook AI Research^39^. It fundamentally augments the BERT model through a series of optimizations and improvements aimed at enhancing the model’s performance and efficiency. Adoma et al.^40^ analyzed the effectiveness of the pre-trained models BERT, RoBERTa, DistilBERT, and XLNet in recognizing sentiment from text, and the results showed that RoBERTa achieved the highest accuracy. Although RoBERTa is less efficient in terms of computational efficiency compared to DistilBERT, considering the seriousness of depression research and the high demand for accuracy, this additional computational cost is acceptable.

Interview data is characterized by significant non-standard language usage, numerous abbreviations, a wealth of emojis, and a diversity of subjects. To process this type of data more effectively, we have chosen the RoBERTa model, which was trained on Twitter tweets, and have meticulously fine-tuned it. This approach is designed to ensure that the model accurately captures the unique features of the interview data, thereby improving the accuracy of analysis and processing.

### Fusion

We performed model integration using the MT5 model and the RoBERTa model, after incorporating a multi-example learning framework. We directly use the average vote to get the final classification result. The formula is as follows:

6 $$\nu_{j}^{i} = \frac{1}{2}\left( {m_{j}^{i} + r_{j}^{i} } \right)$$

where $m_{j}^{i}$ represents the confidence score of the MT5 model for classifying the $j$ instance in the $i$ bag. Similarly, $r_{j}^{i}$ denotes the confidence score of the RoBERTa model for classifying the $j$ instance in the $i$ bag. The final confidence score for classifying the $j$ instance in the $i$ bag is also represented by $\nu_{j}^{i}$.

## Results

### Data preprocessing

**Fig. 5:**
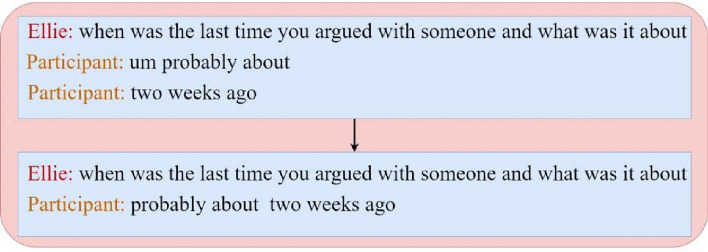
Question and answer data processing diagram.

In the DAIC-WOZ dataset, considering that a participant may provide multiple consecutive answers to a single question, we adopted an integration strategy to more effectively analyze and utilize these responses. Specifically, we merged multiple responses to the same question from a participant into one comprehensive response, as shown in (Fig. 5). Additionally, to ensure that these answers adequately reflect the participant’s genuine intentions and expressions, we sorted the responses based on the number of words they contain, from most to least. This method prioritizes retaining responses rich in emotion.

**Fig. 6:**
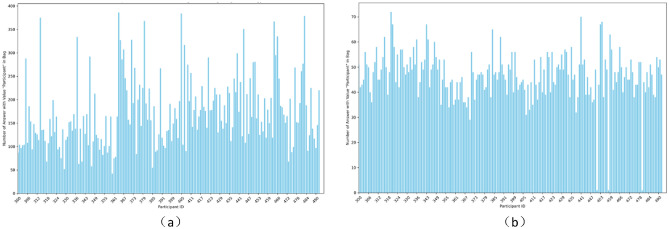
Diagram of the number of answers contained in each package.

The number of answers contained in each package before the integration strategy is shown in (Fig. 6a), and the number of answers contained in each package after the integration strategy is shown in (Fig. 6b). We found that the samples numbered 451, 458, and 480 did not have Ellie’s question text, and there was only one answer in the package, so we did not merge the answers.

For the E-DAIC dataset, we adopt the same integration strategy as the DAIC-WOZ dataset. However, in the E-DAIC dataset, the data has been preliminarily processed, with only the patient’s answers retained. Still, Ellie’s questions exist within the participant’s responses, such as the question “How would your best friend describe you” in the response of participant No. 300. In response to the above issue, we compare the text data of the two datasets and remove the data in the E-DAIC dataset that still does not belong to the participants’ answers. We are not merely using participants’ response data, but are transforming very important questions into participant’s responses. For example, when asked if the participant had been diagnosed with depression before, the respondent would answer “yes” or “no”. This is a very important question, but if we only use the longest answer, this question will be ignored by us because there is only one word in the answer. Therefore, we integrate this Q&A into one sentence. If the participant answers “no”, then we change the response to: “I am healthy and have never tested positive for mental illness such as depression.”; if the participant answers “yes”, then we change the response to: “I was unfortunate enough to be diagnosed with depression.”

### Experiment

#### Experimental environment

All experiments are carried out on a Windows operating system, using an NVIDIA RTX 4070Ti and implemented with the PyTorch framework. The batch size of the training subset is set to 128, learning rate is 0.001 and the number of epochs is defined as 100. The Dropout layer utilizes Monte Carlo Dropout, a method specifically suited for Dropout layers in the field of deep learning for efficiently assessing prediction uncertainty. This technique involves performing multiple (in our case, 10) independent forward passes on the same input feature and computing the variance of these prediction results to estimate the uncertainty of each sample. In the evaluation process of our model, the introduction of Monte Carlo Dropout is primarily aimed at significantly enhancing the detection accuracy and reliability for atypical or difficult-to-identify cases.

#### Evaluation metrics

For depression detection tasks, the common evaluative metrics used to assess the performance of depression detection models include Accuracy (Acc), Precision (P), Recall (R), and F1-score (F1), where the model’s predictions are compared with whether the interviewees truly suffer from depression. The formulas are as follows:

7 $$Acc = \frac{TP + TN}{{P + N}}$$

8 $$P = \frac{TP}{{TP + FP}}$$

9 $$R = \frac{TP}{{TP + FN}}$$

10 $$F1 = \frac{2 \times P \times R}{{P + R}}$$

where TP denotes correctly predicting positive outcomes, FP signifies incorrect prediction of positive outcomes, TN denotes correctly predicting negative outcomes, and FN represents incorrectly predicting negative outcomes.

#### Comparative experiment

Table 2 presents a comprehensive comparison between our proposed method and recent text-based depression detection methods, specifically on the DAIC-WOZ dataset. Our method shows superior performance in terms of Accuracy (Acc), F1 score, Precision (P), and Recall, achieving scores of 0.89, 0.88, 0.89, and 0.86, respectively. Table 3 provides the results of the model on the E-DAIC dataset, highlighting its effectiveness across different datasets.

**Table 2:** Comparison of this method with other methods in the DAIC-WOZ dataset, where the highest scores are highlighted in bold.

Model	Test set			
	Acc	F1-score	P	Recall
Xue et al.^8^	–	0.85	0.79	**0.92**
Burdisso S et al.^6^	–	0.84	–	**–**
Villatoro-Tello E et al.^41^	–	0.8	0.78	0.87
Burdisso S et al.^42^		**0.90**		
Zhang et al.^9^	**0.89**	0.87	0.8	0.85
Ours-multi-MT5	0.83	0.82	0.84	0.79
Ours-multi-RoBERTa	0.85	0.83	0.85	0.80
Ours-multi-MTRB	**0.89**	0.88	**0.89**	0.86

**Table 3:** Comparison of this method with other methods on the E-DAIC dataset, where the highest scores are highlighted in bold.

Model	Test set			
	Acc	F1-score	P	Recall
Villatoro-Tello E et al.^41^	–	0.64	–	**–**
Burdisso S et al.^6^	–	0.84	–	**–**
Xu X et al.^43^	–	**0.86**	0.83	**0.89**
Ours-Multi-MT5	0.80	0.76	0.72	0.81
Ours-Multi-RoBERTa	0.75	0.82	0.85	0.80
Ours-Multi-MTRB	**0.82**	**0.86**	**0.91**	0.82

From Tables 2 and 3, we can see that our fused Multi-MTRB model improves a lot over the results of other researchers. Although there is still room for improvement in some metrics, collectively my model has a better and more stable performance.

#### Interpretability

**Fig. 7:**
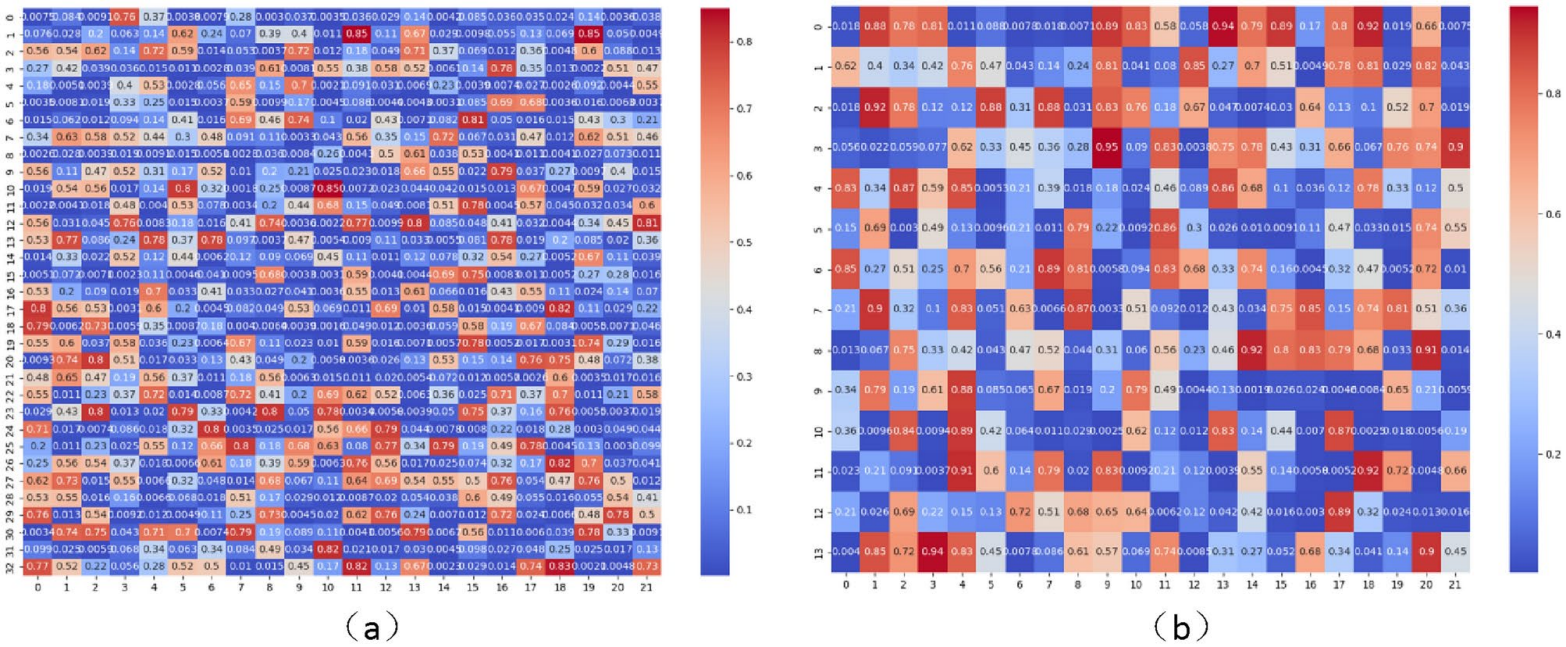
Overall instance depression infographic for the DAIC-WOZ test set.

To intuitively display the effectiveness of the MIL framework employed in this method, and to enhance the algorithm’s interpretability through MIL, Fig. 7 showcases examples from the test sets of the DAIC-WOZ and E-DAIC datasets. These examples include a visual representation of the depression scores for each data bag, facilitating a clearer understanding of how the model processes and interprets data to determine depressive tendencies.

Figure 7 depicts the instance depression graphs from the test set of DAIC-WOZ. Specifically, Figure (a) shows the heatmap of non-depressed individuals’ instances, while Figure (b) presents the depression heatmap of depressed patients’ instances. From Figure (a), we can observe that almost no instances have a depression score exceeding 0.9, whereas in Figure (b), there are numerous instances with higher depression scores. This observation provides an important reference for introducing the hyperparameter α. Furthermore, Figure (a) reveals that there do exist texts containing depressive information in the responses of non-depressed individuals, albeit in significantly lower quantities compared to the number of depressive instances in depressed patients. Hence, the necessity of the hyperparameter β becomes evident.

**Fig. 8:**
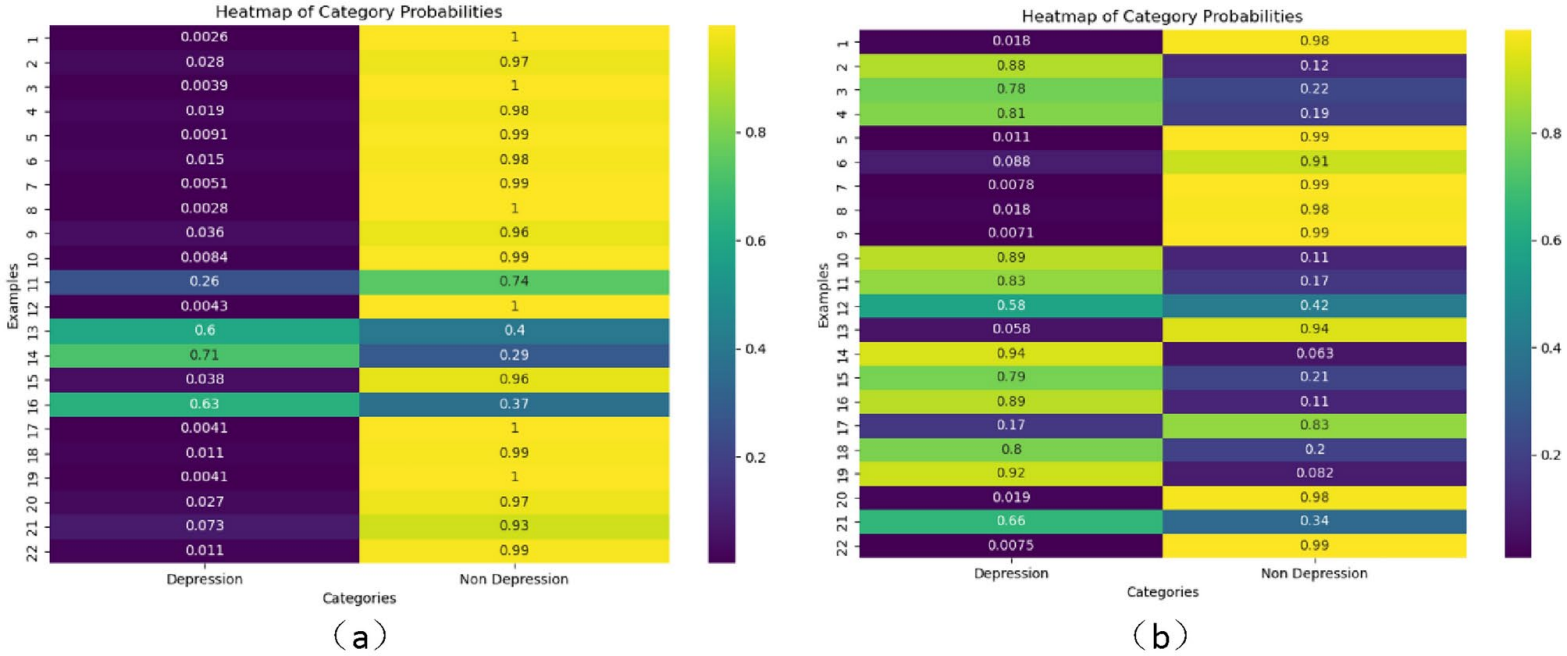
Instance depression information graph.

We extracted the depression information sample graphs of participant numbers 361 and 308 from the DAIC-WOZ test set because their PHQ_Score are 0 and 22 respectively. This indicates that the visual contrast of their depression information is the strongest, the results are shown in (Fig. 8).

**Fig. 9:**
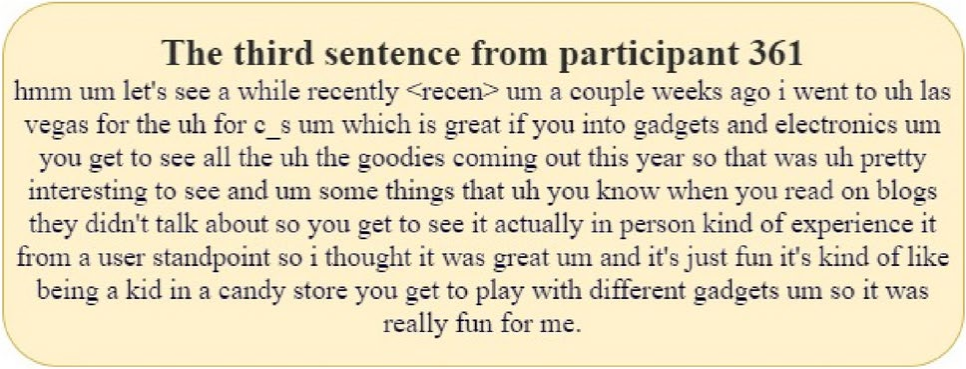
The third sentence of participant number 361.

In Fig. 8, the left image (a) is an example of the depression information of participant number 361. This figure provides us with critical information for the assessment of depression. From the heat distribution in the figure, most examples do not show significant features of depression, which suggests a lack of obvious depressive information in these examples. Based on this observation, we can infer that this participant does not suffer from depression. Furthermore, the participant’s PHQ_Score (Patient Health Questionnaire score) is 0, which confirms that the participant does not exhibit symptoms of depression, thereby further validating our inference. Below, we present the third sentence indicating a 100% probability of non-depression for this participant (as shown in Fig. 9). From the perspective of a normal person, this sentence reflects that the participant is very positive, rather than a depressed patient.

**Fig. 10:**
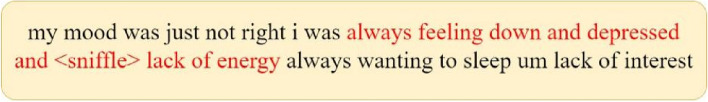
Second sentence of participant number 308 (containing 88% depressive information).

**Fig. 11:**
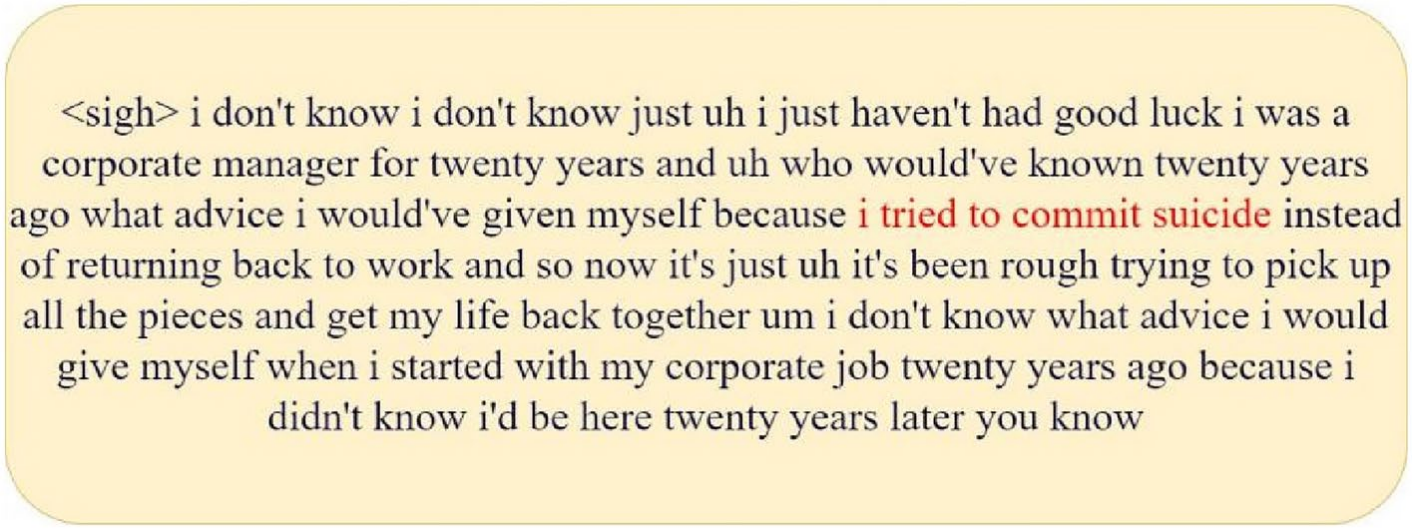
Fourteenth sentence of participant number 308 (containing 94% depressive information).

In Fig. 8, the image on the right (b) is an example of the depression information of participant number 308, forming a sharp contrast with the former. From the heat distribution in the figure, most examples exhibit obvious features of depression, indicating that these examples contain rich depressive information. Therefore, we consider that this participant has severe depression. Similarly, the participant’s PHQ_Score is 22, a high score that indicates the participant has severe depressive symptoms, further validating our inference. By comparing the depression information example figures and PHQ_Scores of the two participants, we can gain a clearer understanding of the manifestations and assessment process of depression. Below, we present two sentences with high depression scores for this participant (as shown in Figs. 10 and 11). From these sentences, especially those in red, it is evident that the participant often feels “depressed” and even has suicidal thoughts. This demonstrates that the participant has a very strong tendency towards depression, proving that our proposed depression detection system has strong interpretability.

**Fig. 12:**
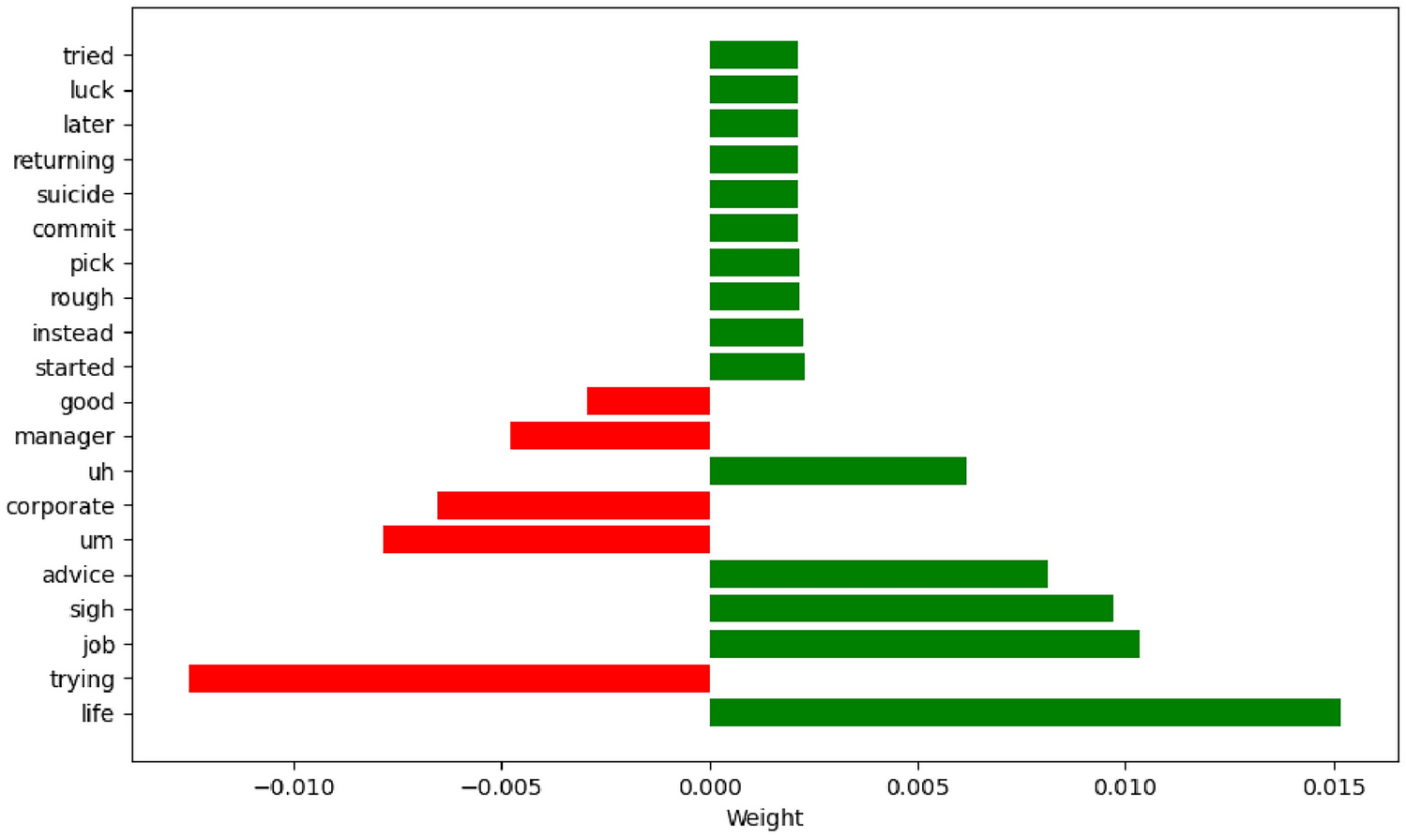
Characteristic contribution map for negative examples.

Although we can focus interpretability down to the sentence level through a multi-instance learning framework, this is not deep enough. Therefore, we introduce the LIME (local interpretable model-agnostic explanations) technique to provide further explanation for negative examples. LIME is a machine learning interpretability technology designed to offer intuitive and easily understandable explanations for the predictions of any given model. The explanations generated by LIME reveal which features contribute the most to the prediction outcomes, as well as whether their contributions are positive or negative, helping users to understand the model’s decision-making process and to increase the model’s credibility and transparency. We conducted a LIME analysis on the negative example sentences displayed in Fig. 11, and the results are shown as follows:

From Fig. 12, it is obvious to observe that the word ‘life’ contributes most significantly to the model prediction results, which implies that there may be some significant differences in the daily life of depressed people compared with normal people, which is in line with our common sense. Meanwhile, the words ‘tired’ and ‘trying’ reveal that depressed people may show different emotional states and behavioural patterns when coping with daily life affairs compared to normal people. These findings provide useful perspectives for understanding the psychological and behavioural characteristics of depressed people.When we consider incorporating the multi-instance learning approach, this technique can more accurately capture and pinpoint the depressive information of the participant. By comprehensively analyzing multiple instances, we can gain a more holistic understanding of the participant’s condition, thereby improving the accuracy of the diagnosis. More importantly, MIL not only enhances diagnostic efficiency but also provides better interpretability. This allows doctors or researchers to clearly understand the contribution of each instance in the diagnostic process, further enhancing the credibility and reliability of the diagnosis.

### Ablation experiment

#### Comparison with different pooling strategies

**Fig. 13:**
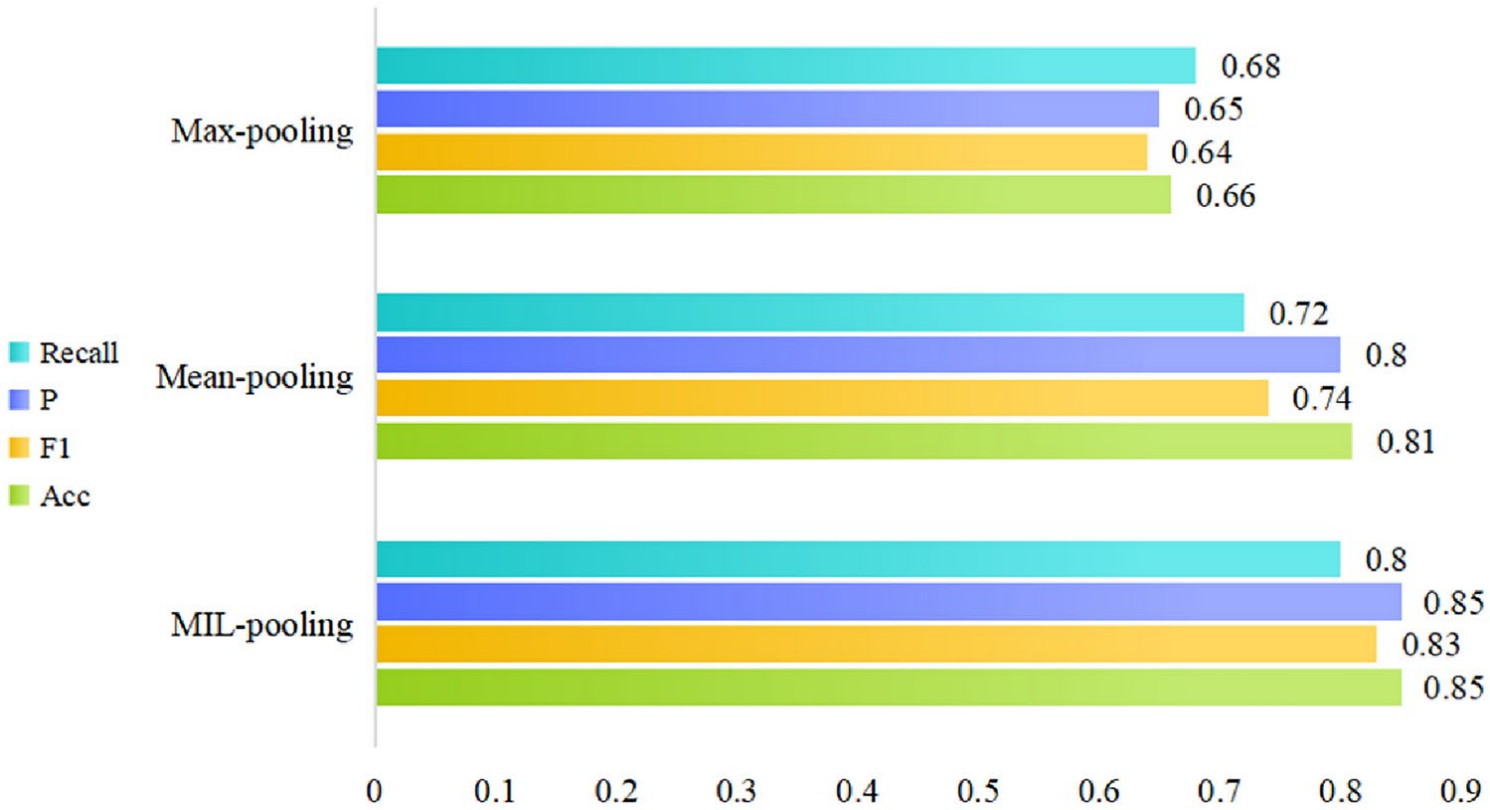
Multiple instance comparison chart.

In this section, to conduct a more granular evaluation of the improved aggregation strategies within the MIL framework, we set up a series of comparative experiments. Specifically, we compared the pooling layers in the RoBERTa and MT5 models with threshold improvements to traditional pooling methods. As shown in Fig. 13, the F1 score of MIL-pooling is improved by 0.09 and 0.19 compared to Max-pooling and Mean-pooling, respectively. While Max-pooling can capture the most prominent features in the sequence, it often ignores other information that may be equally important for the task. In contrast, Mean-pooling considers all features but may weaken the significance of key features due to averaging. Compared to these, pooling layers with threshold optimization (MIL-pooling) can dynamically weight features based on their importance, preserving key features while also taking into account global information to a certain extent, resulting in a significant performance improvement.

#### Effect of depression detection performance with multi-instance learning framework

In this section, we designed an experiment based on the DAIC-WOZ dataset, aiming to deeply explore the performance differences between models applying the multi-instance learning framework and those not applying it in text-based depression detection tasks. We used MT5 and RoBERTa as the base models for performance testing, comparing the experimental results of whether multi-instance learning was incorporated into these two models. Through this design, we aimed to comprehensively evaluate the effectiveness and superiority of integrating the multi-instance learning framework in text data processing. The final results of the experiment are detailed in Table 4.

**Table 4:** Comparison of Model Performance with and without MIL.

Model	Test set			
	Acc	F1-score	P	Recall
MT5	0.74	0.62	0.71	0.61
Multi-MT5	0.83	0.82	0.84	0.79
RoBERTa	0.79	0.70	0.70	0.69
Multi-RoBERTa	0.85	0.83	0.85	0.80

From Table 4, we can observe that the performance of the T5 model (MT5) has been significantly enhanced after introducing the multi-instance framework. Specifically, the evaluation metrics show notable improvements: the accuracy (Acc) increases by approximately 0.1, the F1 score rises by 0.2, the precision (P) improves by 0.13, and the recall grows by 0.18. Similarly, when we adopt the multi-instance framework in the RoBERTa model, the model’s performance also experiences significant enhancement: the accuracy (Acc) increases by 0.07, the F1 score rises by 0.13, the precision (P) improves by 0.15, and the recall grows by 0.11. These improvements indicate that the introduction of multi-instance learning has a positive and significant impact on the overall performance of the models.

The significant effect of multi-instance learning in pre-trained models mainly stems from its ability to effectively “shorten” sentence lengths, which is crucial for handling long text tasks. When we input participants’ responses into pre-trained models, we often face a challenge: how to ensure that the sentence lengths comply with the model’s limitations, such as the common 512-token limit. Overly long sentences can not only lead to inefficient model processing but may also result in the loss of important information due to truncation. However, by using the multi-instance method, we can split the response set into multiple shorter sentences or fragments, ensuring that each fragment’s length is less than 512 tokens. This approach not only avoids semantic loss caused by sentence truncation but also enables the model to more accurately capture the key information within each fragment. Therefore, the multi-instance method has significant advantages in handling long text tasks, effectively enhancing the performance of pre-trained models.

#### Hyper-parameters ${\varvec{\upalpha}}$ and ${\varvec{\upbeta}}$

In this study, the hyper-parameter α represents the threshold at which a single instance within a bag can directly determine the bag as a negative bag. The hyper-parameter β represents the threshold for the presence of negative instances within a positive bag. In text sentiment analysis, especially when dealing with interview data, interviewees may inevitably provide responses that do not reflect their actual situation due to their emotions. This means that a positive class (or positive bag) may contain negative instances. This mixture, as shown in Fig. 3, can negatively impact the accuracy of our analysis. To effectively address this challenge, we introduced the hyper-parameter $\upbeta$ as a solution. Essentially, this hyper-parameter represents a threshold to determine the extent to which the presence of negative instances in a positive bag can be tolerated, ensuring that our model remains more precise and reliable during the analysis process.

**Fig. 14:**
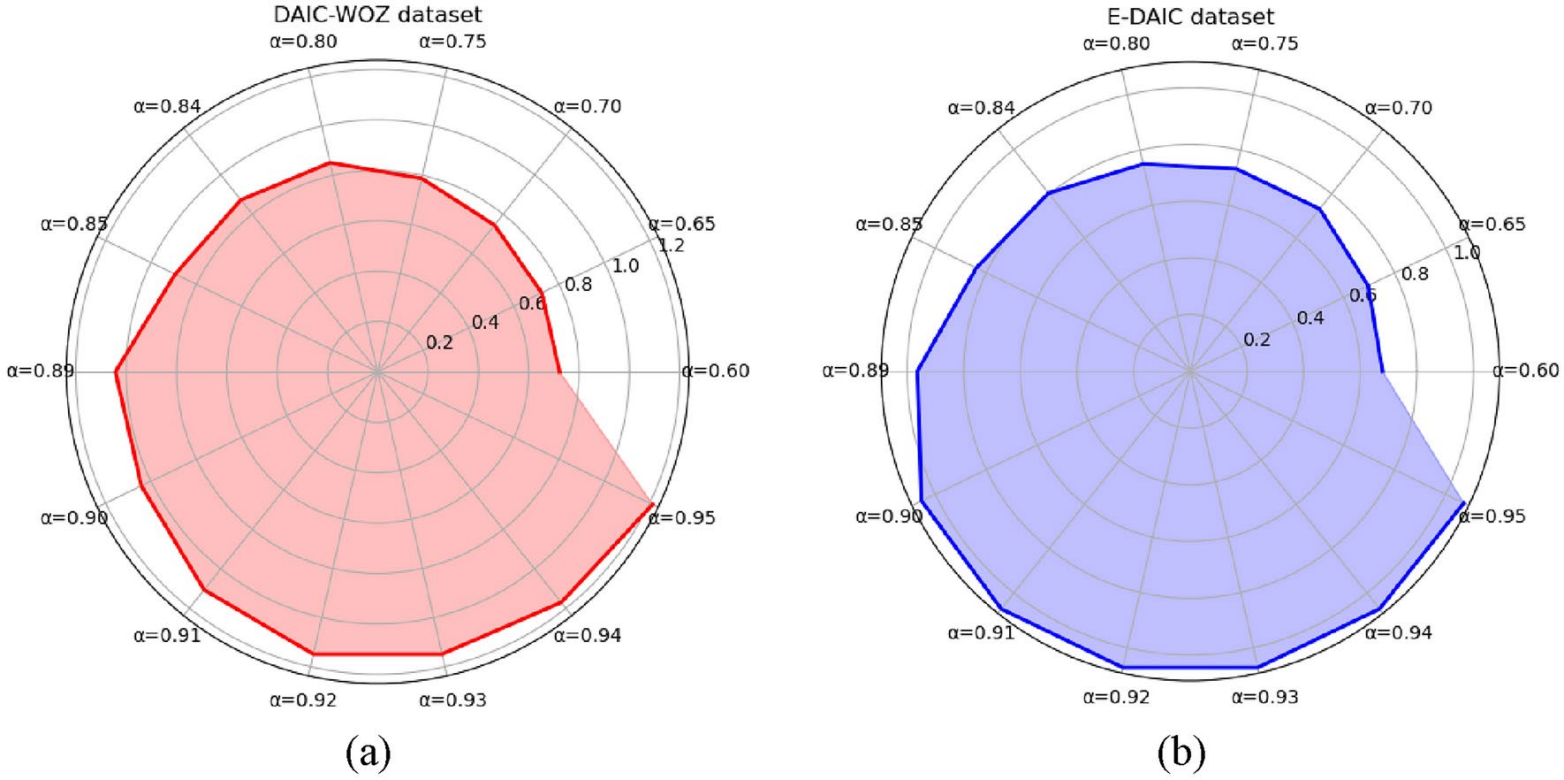
Changes in accuracy (Acc) with Hyperparameter α.

Figure 14 illustrates the variation of the accuracy (Acc) metric with different values of the hyperparameter α. The left-side graph (a) shows the changes in the DAIC-WOZ dataset, while the right-side graph (b) represents the changes in the E-DAIC dataset. On the DAIC-WOZ dataset, when the hyperparameter α is set to 0.95, the Acc metric reaches its highest value of 0.78. On the E-DAIC dataset, when the hyperparameter α is set to 0.91, the Acc metric reaches its highest value of 0.71. Additionally, from the radar chart, we can observe that after the Acc metric reaches its peak, it remains unchanged as the hyperparameter α increases. This indicates the presence of instances with very high depression scores within the bags, and assigning higher weights to these instances is reasonable.

**Fig. 15:**
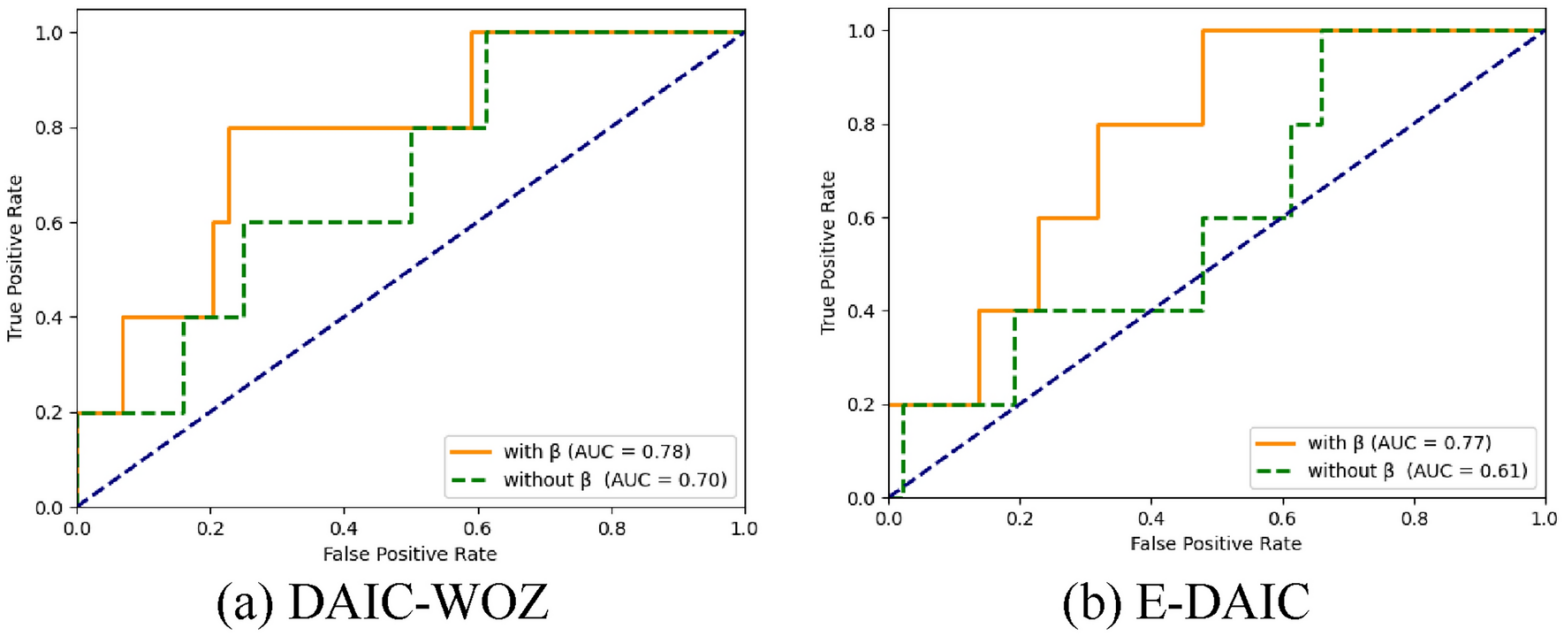
ROC curve graph. (**a**) DAIC-WOZ, (**b**) E-DAIC.

After determining the optimal value for α, we considered that some participants might have mild depression, meaning that their responses contain depressive information but with a low depression score. Additionally, even if participants do not have depression, they might believe they are depressed, leading to responses mixed with depressive information. To address these two issues, we conducted experiments regarding the hyperparameter $\upbeta$. To assess the overall performance of the model more effectively, we have incorporated the receiver operating characteristic (ROC) curve and the Area under the curve (AUC) metrics. The ROC curve demonstrates the classifier’s performance at various threshold levels by plotting the False Positive Rate (FPR) on the x-axis and the True Positive Rate (TPR) on the y-axis. The closer the ROC curve is to the upper left corner, the better the classifier’s performance. The AUC (Area Under the Curve) is the area beneath the ROC curve and is used to quantify the overall performance of the classifier. The experimental results are shown in the table below:

As can be seen from the experimental results in Table 5, the hyperparameters are crucial to the effectiveness of the model. On the DAIC-WOZ dataset, the accuracy (Acc) of the model increased by 6% and the F1-score by 5%, while on the E-DAIC dataset, the accuracy also increased by 5% and the F1-score by 6%. This also indicates that some participants may have been suffering from early depression. Figures 15a and b respectively display the ROC curve comparison before and after applying parameters $\upbeta$ on the DAIC-WOZ and E-DAIC datasets, respectively, where the AUC is 0.78 on the DAIC-WOZ dataset, and the result is 0.77 on the E-DAIC dataset. From these charts, it is clearly observable that, on both datasets, the ROC curves exhibit a trend of shifting towards the left after parameters are introduced. This phenomenon provides strong evidence that, in the task of text sentiment analysis, positive bags indeed contain instances with negative sentiments.

**Table 5:** Comparison of models with and without hyperparameter $\upbeta$.

Model	Acc		F1	
	DAIC-WOZ	E-DAIC	DAIC-WOZ	E-DAIC
Without $\upbeta$	0.79	0.71	0.78	0.76
With $\upbeta$	0.85	0.75	0.83	0.82

## Discussion and limitation of our work

This paper is the first to apply the multiple instance learning (MIL) framework to the field of textual interview data in order to address the problems of inadequate text representation and ineffective information extraction in long texts. By performing independent feature extraction for each instance under the MIL framework, this study not only improves the overall text representation capability, but also alleviates the sample imbalance problem in the dataset. In addition, this paper improves the previous aggregation strategy by introducing two hyperparameters to accommodate the uncertainty in the text sentiment domain. The constructed integrated model of MT5 and RoBERTa (called Multi-MTRB) is capable of extracting features from each instance and outputting a confidence score indicating the presence of depression information.

Although we achieved relatively good results, our work is not without limitations. Firstly, although multi-instance learning can segment text data and mitigate the problem of class imbalance, it cannot be ignored that we should seek better solutions. Secondly, our research is based on the DAIC series datasets: DAIC-WOZ and E-DAIC. These two datasets are limited in size and cannot represent a broader population, which restricts the generalizability of our findings across different cultural backgrounds. Lastly, we have conducted minimal error analysis on the model, which is indeed vital for the continuous improvement of the model and its application in real-world clinical settings. Therefore, it is essential to further discuss the errors produced by the model, specifically false positives and false negatives.

### Error analysis and model improvement

Initially, we noted that the model’s errors are not randomly distributed but are associated with specific data characteristics. Specifically, the following is an analysis of the model’s misclassification scenarios:

#### False positive analysis

We observed that the model tends to generate false positives in certain types of interview responses. These responses may contain specific linguistic patterns or themes that are associated with depression but do not necessarily indicate that the interviewee actually suffers from clinical depression. As shown in Fig. 3, the false positive case highlights a crucial issue: although the patient’s responses are laden with intense emotions and resemble depressive symptoms, under the multi-instance framework, the system hastily diagnoses the patient with depression based solely on one or more examples exhibiting significant depressive emotional features, leading to the misclassification of Participant 302, who is not actually depressed, and resulting in a false positive. To effectively avoid such misjudgments, we recognize that the construction of a depression detection system should not be confined solely to text modality, but rather should be a system that integrates multi-modal data. Specifically, beyond text information, modalities such as electroencephalography (EEG) and audio should also be included. These additional data points contribute to a more comprehensive assessment of the patient’s emotional state, thereby reducing the occurrence of false positives due to erroneous interpretation of textual emotions. Furthermore, we should explore and adopt more advanced uncertainty models. Although current research has attempted to introduce Monte Carlo Dropout (MCD) technology to address uncertainty issues, its improvement effect is still limited. Therefore, in the future, we should strive to develop and apply more precise and efficient uncertainty strategies to further enhance the accuracy and reliability of depression detection. By integrating multi-modal data and optimizing uncertainty models, we can build a more robust and accurate depression detection system, providing patients with more reliable diagnostic services.

#### False negative analysis

Conversely, the model is more likely to produce false negatives when dealing with responses that are implicitly expressed or have less apparent symptoms. This may be due to the weak depressive signals in these responses, which the model has not yet been able to effectively capture. To improve the model in a targeted manner, we propose the following strategies:

#### Feature analysis

Further analyze the textual features that lead to misclassification, including vocabulary usage, sentence structure, and emotional expression, to better understand the model’s decision-making process.

#### Data augmentation

Based on the results of the error analysis, augment the training data by adding samples related to misclassification to enhance the model’s robustness.

### Ethical considerations in clinical deployment

Although our study has confirmed ethical approval and data anonymization, the broader ethical implications of deploying our model in real-world clinical settings require careful consideration. A key aspect is the management of false positives and false negatives, which can have significant consequences in a clinical environment. Our discussion must extend to how such misclassifications can affect patient care. For instance, false positives may lead to unnecessary anxiety and treatment, while false negatives could result in delayed or missed treatment opportunities. Therefore, we emphasize the importance of human oversight: in any case, the model’s decisions should be subject to the supervision and ultimate judgment of clinical experts. It is crucial to ensure that during the model’s decision-making process, human experts can provide personalized humanitarian care based on the specific circumstances of the patient, and that the model is used to support rather than replace clinical decision-making. After the model is deployed, its performance should be continuously monitored, and its clinical applicability and ethical impact should be regularly evaluated to ensure patient safety and the quality of care.

### How clinical doctors integrate into the workflow

Integrating depression detection models into clinicians’ workflows in clinical practice is a complex and meticulous process. This process not only requires clinicians to have sufficient understanding and trust in the models, but also necessitates their continuous adjustment and optimization of their working methods in practice to ensure that the models can truly play an auxiliary role, improving diagnostic efficiency and accuracy.

Firstly, clinicians need to clarify the role of the models in clinical decision-making. Depression detection models serve as auxiliary tools that can help doctors identify potential depressive symptoms more quickly and accurately. However, the models cannot replace doctors’ professional judgment and experience. Therefore, when using the models, doctors need to maintain a cautious and rational attitude, carefully interpret the output results of the models, and make comprehensive judgments based on the patients’ actual conditions and clinical manifestations.

During the integration process, clinicians also need to pay attention to communicating with patients. Since the use of models may involve patients’ privacy and data security issues, doctors should use the models after informing patients and obtaining their consent. Meanwhile, after the models output results, doctors need to fully communicate and explain to patients to ensure that they understand the diagnosis and accept the corresponding treatment plan.

Furthermore, clinicians need to continuously adjust and optimize their working methods in practice. Since each patient’s situation is unique, the models may make misjudgments or omissions in some cases. Therefore, doctors need to remain vigilant when using the models, promptly identify and correct their errors. At the same time, doctors also need to flexibly adjust the use of models based on their own experience and judgment to ensure that the models can better adapt to the needs of clinical work.

## Conclusion and future work

In the field of depression detection based on text interview data, this study has achieved remarkable results by incorporating the multi-instance learning framework into pre-trained models and integrating the RoBERTa and MT5 models. Comparisons between models with and without the multi-instance learning framework indicate that multi-instance learning can address the challenge of accurately capturing emotional information in long texts. Finally, we also considered the impact of negative instance noise in positive bags and introduced two hyperparameters to mitigate these effects. This study not only provides guidance for Automatic Depression Detection (ADD) tasks but also offers valuable insights and new directions for text sentiment analysis.

In the future, we will augment our model mechanisms to capture temporal or sequential dependencies, striving to explore the significance of time and response order dependency in clinical detection of depression and gain a deeper understanding of the dynamic processes of depression. Additionally, we plan to integrate electroencephalogram (EEG) data with acoustic features and textual expressions for comprehensive analysis. This approach demonstrates immense potential as a more comprehensive and accurate screening tool for depression, which not only facilitates early identification and diagnosis of depression but also significantly enhances the effectiveness of treatment and intervention, thereby markedly improving patients’ medical satisfaction and overall quality of life. Lastly, addressing the current issue of insufficient interpretability of models in depression, we intend to combine depression knowledge (in the form of knowledge graphs) with deep learning methods to construct interpretable depression detection models. This will be a highly practical and meaningful direction.

## Data Availability

The DAIC-WOZ dataset is publicly available at (https://dcapswoz.ict.usc.edu/). The E-DAIC dataset is publicly available at (https://dcapswoz.ict.usc.edu/).
